# Editorial: Monitoring and risk assessment of pesticide residues and mycotoxins - A potential public health concern

**DOI:** 10.3389/fpubh.2023.1293726

**Published:** 2023-11-16

**Authors:** El-Sayed A. El-Sheikh, Mohammad D. H. Prodhan, Sivaperumal P

**Affiliations:** ^1^Department of Plant Protection, Faculty of Agriculture, Zagazig University, Zagazig, Egypt; ^2^Pesticide Research and Environmental Toxicology Section, Entomology Division, Bangladesh Agricultural Research Institute, Gazipur, Bangladesh; ^3^Chemical Science Division, ICMR – National Institute of Occupational Health, Ahmedabad, Gujarat, India

**Keywords:** pesticide residues, mycotoxins, monitoring, risk assessment, public health

Pesticides are widely used to control various pests, including insects, fungi, and weeds, and are also used as plant growth regulators. According to the Food and Agriculture Organization, the global use of pesticides exceeded 2.5 million tons in 2020 (1). Although the use of pesticides leads to improve the quantity and quality of agricultural products, their intensive and unconscious use leads to environmental pollution and health risks (2). For example, pesticide residues in food, soil, and the environment in general represent a serious problem due to their persistence, bioaccumulation, and toxicity (3). Previous studies have shown the presence of concentrations of chemical pesticides higher than the permissible limits in vegetables and fruits in many countries, including China, which is considered one of the largest consumers of pesticides in the world. The presence of pesticides in agricultural crops usually affects the export of their products (Ma et al.). Contamination of food with pesticides poses potential risks to consumers if eaten without treatment. It has been found that eating food that contains pesticide residues, especially those that exceed the permissible limits, can cause cancer, heart disease, and other diseases to some extent. Studies also confirm that there are negative effects when consumers are exposed to low doses of pesticides over a long time, which include birth defects, low birth weight, and fetal death.

Mycotoxins arise in the field or during storage due to moisture and temperature. They are secondary derivatives of filamentous fungi and include aflatoxin and ochratoxin A (OTA). Aflatoxin and OTA contaminate many food stuffs such as grains, legumes, nuts, spices, herbs, coffee, cocoa beans, etc. *Aspergillus flavus* and *A. parasiticus* are the main sources of producing aflatoxin, while *A. ochraceus, A. carbonarius, A. niger*, and *Penicillium verrucosum* are producing OTA. There are many types of aflatoxins, and type B1 (aflatoxin-B1) is considered the most powerful among all mycotoxins, which has been classified by the International Agency for Research on Cancer (IARC) as a human carcinogen (Group 1) due to its effect on vital organs in the body such as liver. Studies have indicated that aflatoxin-B1 may be the main cause of various poisoning cases (acute, chronic, genetic, and immunotoxic), in addition to being a teratogenic substance. Although OTA is less toxic than aflatoxin-B1, the IARC has classified it as a probable human carcinogen (Group 2b), where it can cause many health consequences such as nephrotoxicity, hepatotoxicity, neurotoxicity, teratogenicity, and immunotoxicity. Recent studies in Lebanon have shown that the consumption of herbs and nuts, especially spices, can lead to an increase in health risks associated with aflatoxin-B1 and OTA, and the probable reason for this situation may be due to poor storage conditions (Daou et al.).

The number and distribution of pesticide and mycotoxin poisoning cases can be obtained from the health surveillance system, but it often does not provide information about the risks of exposure to these toxins and the causes of poisoning. Investigating pesticide residues and mycotoxins, in various elements of the environment, such as food, water, soil, and others, using traditional or modern methods, has a vital role in monitoring and identifying the risks of these pollutants and managing them. Therefore, many governments are setting rules and regulations to reduce the use and risks of pesticides based on these studies. The article by Sapbamrer et al. reviews the role and current status of the Government of Thailand in reducing the use and risks of pesticides, and proposes several recommendations in order to promote sustainable development in Thailand. Accordingly, several policies have been put in place to reduce the use of pesticides and their risks, such as amending some acts related to hazardous substances, consumer protection, and the act on combating occupational and environmental diseases. Also, recommends using good agricultural practices, organic farming, reducing the use of highly toxic pesticides, and applying the maximum residue limit in food. With regard to limiting the use of pesticides inside the home to combat insects that transmit diseases, the Ethiopian study carried out by Mekasha et al. indicates the need to strengthen the dissemination of health information about indoor residual spraying (IRS) to control mosquito that transmit malaria. For this reason, their study suggested that to increase the level of acceptance of IRS among communities, health interventions and services should focus on creating awareness about the effectiveness of IRS, the suitable time/season of spraying, and the side effects of IRS. Therefore, enhanced dissemination of health information can help encourage acceptance of IRS in order to reduce pesticide use and risks.

Therefore, the purpose of this Research Topic was to develop a reference for the latest high-quality publications in the field of monitoring and risk assessment of pesticide residues and mycotoxins through the estimation of their residues in various food components. Which, in turn, show the types of food and sites of production (rural or urban) that entail potential risks when consumed? In addition, the Research Topic explored the role that governments can play in strengthening the dissemination of health information regarding the application of pesticides in disease-transmitting insect control. Hence, it provides necessary information for decision makers as a reference on the status of pesticide residues and mycotoxins with the aim of supervising and managing them in order to reduce the use and risks of them.

## Author contributions

EAE: Conceptualization, Investigation, Methodology, Visualization, Writing—original draft, Writing—review & editing. MP: Conceptualization, Investigation, Methodology, Visualization, Writing—original draft, Writing—review & editing. SP: Conceptualization, Investigation, Methodology, Visualization, Writing—original draft, Writing—review & editing.
